# Genetics of autosomal recessive intellectual disability

**DOI:** 10.1007/s11825-018-0209-z

**Published:** 2018-10-24

**Authors:** Rami Jamra

**Affiliations:** Institute of Human Genetics, University Medical Center, Philipp-Rosenthal-Str. 55, Leipzig, Germany

**Keywords:** Exome sequencing, Pleiotropy, Heterogeneous, Consanguineous, Neurodevelopmental disorders, Low functional autism, Exom-Sequenzierung, Pleiotropie, Heterozygot, Konsanguin, Entwicklungsverzögerung, Low-Functioning Autismus

## Abstract

In the last few years, next-generation sequencing has led to enormous progress in deciphering monogenic forms of intellectual disability. Autosomal dominant intellectual disability (ADID) and X chromosomal intellectual disability (XLID) have been the focus of research. Apart from metabolic disorders, autosomal recessive intellectual disability (ARID) is still behind, probably because it is more heterogeneous and less prevalent in industrial populations. The prevalence of ARID in a cohort of affected children of an outbred population is estimated to be about 10%, with an upward tendency in still unclarified cases. The risk for ARID in children of first cousins or closer is a magnitude higher than for children of unrelated parents. Taken together, it seems that children of related parents are at a 2 to 3 times higher risk for ID. There are no prevalent ARID genes, pathways, or protein complexes and the functions of the affected proteins are very diverse and limited not only to neurological aspects. Thus, in a regular case, there is no reasoning for picking a few genes for a first diagnostic step, and a genetic diagnosis of ID in general, and ARID specifically, is better made using large panels or exome sequencing. In addition, in the last few months, evidence has been growing that many ARID genes are pleiotropic and that the resulting phenotypes may have a broad spectrum. For an exhaustive deciphering of the genetics of ARID, we suggest research at the level of single genes rather than large meta-analyses.

## Zusammenfassung

„Next generation sequencing“ (NGS) führte in den letzten Jahren zu enormen Fortschritten bei der Entschlüsselung monogener Formen der geistigen Behinderung. Bisher standen die autosomal-dominante geistige Behinderung (ADID) und die X‑chromosomale geistige Behinderung (XLID) im Mittelpunkt der Forschung. Bis auf die Stoffwechseldefekte ist die autosomal-rezessive geistige Behinderung (ARID) weniger erforscht, wahrscheinlich weil sie heterogener ist und in den Industriebevölkerungen eine geringere Prävalenz aufweist. Die Prävalenz von ARID in einer Kohorte betroffener Kinder einer Outbred-Population wird auf etwa 10 % geschätzt, wobei eine Tendenz nach oben in noch ungeklärten Fällen zu erwarten ist. Das Risiko für ARID bei Kindern von Cousins und Cousinen ersten Grades oder näher verwandt scheint um ungefähr den Faktor 10 höher zu sein als bei Kindern von nichtverwandten Eltern. Nach Berücksichtigung anderer Ätiologien ist das Gesamtrisiko für ID bei Kindern verwandter Eltern etwa 2- bis 3-fach höher. Es gibt keine Gene, Signalwege oder Proteinkomplexe, welche auffällig häufig bei ARID betroffen sind. Die Funktionen der betroffenen Proteine sind sehr vielfältig und nicht nur auf neurologische Aspekte beschränkt. Daher ist in der Regel die Auswahl einiger Gene für einen ersten diagnostischen Schritt nicht gerechtfertigt. Eine genetische Diagnose eines Falles mit ID im Generellen und von ARID im Besonderen ist durch große Panels oder Exom-Sequenzierung zu stellen. In den letzten Monaten hat sich zudem gezeigt, dass viele ARID-Gene pleiotrop sind und die resultierenden Phänotypen ein breites Spektrum aufweisen können. Für eine vollständige Entschlüsselung der Genetik von ARID sind Untersuchungen an einzelnen Genen und weniger die großen Meta-Analysen erforderlich.

In this review, the term intellectual disability (ID) also includes alternative terms such as low functioning autism spectrum disorders, mental retardation, and neurodevelopmental disorders (NDD). Genetic causes of ID are highly heterogeneous, including large chromosomal abnormalities, submicroscopic copy number variants, and monogenic forms due to pathogenic variants in single genes [1, 15, 16]. The monogenic forms are classified based on inheritance mode to X linked (XLID), autosomal dominant (ADID), and, the subject of this review, autosomal recessive intellectual disability (ARID).

## Prevalence of ARID in an outbred population

The DDD study of 7448 ID cases revealed that autosomal recessive defects accounted for 11.7% of all cases with a clear molecular diagnosis, but that an over-proportionate fraction of ARID was in consanguineous families [1]. The DDD data were re-evaluated specifically regarding ARID (not in a  peer-reviewed, but in a publicly available manuscript [7]). Taking the full cohort, including undiagnosed cases, it showed that ARID is rarely diagnosed in outbred populations (in only 3.6% of all ID cases), whereas 50% are diagnosed due to a *de novo* mutation [7]. However, the data still need to be considered with caution, e. g., there is no justification that biallelic mutations were identified in only 12% of cases with more than one affected child. In addition, known and relatively common ARID genes (e. g.. *MAN1B1*) did not pass the significance threshold of the study.

Based on the results of routine panel diagnostics at the Institute of Human Genetics in Leipzig, 26 out of 140 (19%) ID cases that were clarified because of a mutation in a single gene were of autosomal recessive inheritance. In independent studies, the fraction of ARID in diagnosed cases after exome sequencing was described as 6 out of 23, 9 out of 47, 5 out of 12, and 4 out of 14 [19–22], totally a comparable number of 24 out of 96 (25%). However, delineating the observations in Leipzig revealed that of these 26 ARID cases, 11 were identified in families with names that suggest a migration background. Nine of the variants were homozygous, and of these, only one (in *TSEN54*) was identified in a German European family. After limiting the cohort to German Europeans, we ended up with mostly compound-heterozygous variants in 15 ARID genes out of 110 clarified ID cases (14.6%), thus still clearly higher than in the published DDD study. We assume that the cohorts of the other, above mentioned studies may eventually lead to similar numbers.

It must be considered that the above-mentioned numbers of the DDD studies and other data cannot be directly compared owing to differences in methods (clinical examination and criteria of inclusion and testing in addition to sequencing and data analyses; exome vs panel, trio vs solo, and evaluation case per case vs meta-analyses) and to a lack of information in the published data. Thus, taken together, an exact number of ARID cases cannot be given in an outbred population. However, considering all previous numbers and studies together, we estimate that ARID makes up about 10% of all diagnosable ID cases in an outbred population.

## Risk for ARID in a consanguineous family

The DDD studies [1, 7] report that in 50% of the British European cohort [7], a pathogenic *de novo *variant was identified, and that the prevalence of such variants is 6% in individuals with autozygous runs equivalent to a first cousin union or closer [1]. The prevalence of autosomal recessive mutations was 3.2% and 47% for the two cohorts respectively [7].

As the DDD study [1] reports that ID due to a de novo variant is 1:295, diagnosable autosomal recessive forms of intellectual disability in a community with consanguineous mating would be roughly estimated to be about 2% (8 × 1:295, as the prevalence of diagnosable ARID [47%] is almost 8 times higher than that of a diagnosable *de novo* variant 6%), a risk that adds to the basic risk for non-consanguineous mating, making the prevalence of diagnosable monogenic ID in the children of first cousins or closer two timers higher. Other extrapolations from the same study [1] (e. g., based on Fig. 1c) or from other studies (e. g., Reuter et al. [16]) lead to smaller numbers, between 2.5 and 4.5 times more ID that is monogenic and diagnosable in children of consanguineous families. However, not considered in these estimations are nongenetic causes, aneuploidies, *de novo* copy number variants, imprinting defects, and multifactorial causes, in addition to the high number of undiagnosed cases, for which the proportions of different etiologies are still unknown. These factors necessitate a correction of the above estimation, making it plausible that the prevalence of ID is 2–3 times higher in children of consanguineous families. This overlaps with literature on the estimated basic risk for serious congenital and genetic disorders at the age of 1 year of 4–4.5%, which is twice as high as that of an outbred family. Follow-up studies confirmed the doubled risk (8% vs 4%) [5]. It also fits with the results of other epidemiological studies that show that close relation of the parents, double cousin or uncle-niece unions makes ID 3–4 times more common than in children of unrelated parents [4, 18].

## How many ARID genes are there?

Based on SysID [10], there are 684 genes that, when mutated, would lead to an ARID form and 378 autosomal recessive candidate genes, for which only a single patient or single family has been reported (last update of the database in June 2018). Calculations based on the extrapolation of 120 known XLID genes and the size of chromosome X estimated that the number of ARID genes would be about 2500–3000 [8, 11]. Otherwise, authors tend to write that the ARID genes “runs into thousands,” but no exact numbers are given. In the most recent re-evaluation of the DDD study [7], 903 ARID genes clarify roughly half of the observed excess of damaging biallelic genotypes, suggesting that half of the ARID cases might not be clarified. Assuming that the second half would be clarified by more genes (as they are rarer), the figure of further 2000 ARID genes seems plausible.

## Role of ARID in undiagnosed cases

Not included in the paragraphs above is the estimation of ARID in undiagnosed ID. Consideration in this regard should take into account that a) studies rather diagnose haploinsufficiency forms of ADID because of truncating variants and that pathogenic loss-of-function missense variants in addition to gain-of-function variants would still make up a significant number of undiagnosed cases [1], b) ARID is more heterogeneous and despite a large number of known genes, most (two thirds to three quarters) ARID genes have not yet been identified, and c) deciphering noncoding variants and multifactorial inheritance in ID is still far behind. At the Institute of Human Genetics in Leipzig we scientifically evaluated a cohort of 135 clinically negative trio exomes and we identified in 98 cases, 191 candidate variants in 177 genes. Considering only strongly convincing genes in families with a single candidate gene (*n* = 41), 80% of the candidates were de novo, 17% were recessive, and 2% were X‑linked. This experience, combined with the reasoned assumption that most of the ARID genes have not yet been identified, whereas the ADID and XLID genes are better characterized, led to an estimation that the fraction of ARID in unsolved cases would be higher than in now clarified cases, possibly between 15 and 20%. In total, this means that at a time point in the near future, at which all monogenic disorders are identified, ARID would contribute to 10–15% of the cases in an outbred population. It should be noted, however, that the DDD study, which specifically evaluates ARID, rather suggests a less prominent role for ARID in nonconsanguineous cases in cases that are still unclarified [7].

## Are there any prevalent ARID genes?

Based on the data of HGMD and ClinVar, variants in only a few ARID genes seem to be reported frequently, including *GALT*, *VPS13B*, *ASPM*, *SPG11*, *MUT*, *GLDC*, *CEP290*, *POLG*, *LAMA2*, and *SMPD1*. However, this does not represent the true distribution of ARID genes, because several of these genes have a minor/mild presentation without ID and because syndromic forms of ARID (e. g., VPS13B and ASPM) have been known for years and the accumulation of mutations in these genes rather reflects the possibility of a clinical diagnosis. Indeed, in contrast to autosomal dominant forms of intellectual disability, for which some few genes are over-represented (*SYNGAP1*, *ARID1B*, *ANKRD11*, *SCN1A*, and some others [1]), none of the large ARID studies that were performed on over 100 families showed any particularly prevalent gene in ARID [3, 6, 8, 16, 17]. These studies are the five biggest so far, were published in 2017 and 2018, and were performed on cohorts that were recruited in countries with less developed health insurance systems and medical care, thus often not pre-filtered for clinically and metabolically recognizable causes of ARID. The number of examined families in these studies totals 1131 (Harripaul 192, Hu 404, Reuter 152, Riazuddin 121, Anazi 262). Of these, 484 families were diagnosed because of mutations in 334 genes that were already known to cause ARID (numbers after manual curation). Of these, 75 genes showed mutations in more than one family; 10 families with mutations in *VPS13B* (2.3%), 9 families with mutations in *MAN1B1* (2%), and 8 families with mutations in *ADAT3* or *AP4M1*. Taken together, it seems that Cohen syndrome is more prevalent than other forms of ARID, possibly because of the large gene size, but that there is no justification for a gene-specific or panel diagnostic when ARID is suspected.

## Are there any prevalent ARID disease groups?

Some ARIDs are classified to well-described syndromes such as ciliopathies (Joubert syndrome, Bardet–Biedl syndrome, and others), metabolic disorders, mitochondriopathies, leukodystrophies, and other entities and groups. However, the prevalence of each group in the total number of ARID cases is low, e. g., evaluating the 32 Joubert syndrome genes in OMIM in 1131 ARID families in five large studies (see also the previous paragraph) revealed mutations in 9 genes in 14 families (1.2% of examined families). In the same cohort, there is only one mutation in 17 genes for the autosomal recessive hypomyelinating leukodystrophy, and only four mutations for Bardet–Biedl syndrome. There are no reliable data on metabolic disorders in ID, but in general, the yield of metabolic studies in cases of ID is low and varies from 0.2 to 8.4%, with a median of 1% [9]. Indeed, all studies conclude with the final statement that genes mutated in ARID are highly heterogeneous and belong to different pathways and tissues [6, 8, 16].

The lack of common genes and pathways in ARID specifically (and in ID in general) results in the recommendation that in the absence of a clear syndromic differential diagnosis (e. g., Cohen syndrome), after having basic clinical diagnostic tests, there is no justification to run exhaustive imaging, metabolic testing, or testing regarding differential diagnostics, as panel or exome genetic testing would be timely and far more economical.

Still, the retrospective classification of ARID based on pathomechanisms and pathways is relevant. Having different mutations in different genes that lead via the same mechanism to the phenotype may offer a starting point for therapy.

## Syndromic versus nonsyndromic ARID

Five to ten years ago, the first reports were published that identified causes of ID in so-called nonsyndromic forms of ARID. The main justification of the term “nonsyndromic” was the absence of clear and recognizable symptoms that would help to set a diagnosis based on the clinical presentation. Indeed, many of the cases were rather unspecific and several ID forms that were reported initially to be nonsyndromic turned out to be syndromic, as other cases with overlapping phenotypes have been identified [2]. More important is being aware of variable presentations as there is recently accumulating evidence that the spectrum of symptoms due to bi-allelic mutations in established ARID genes may vary enormously.

## ARID and pleiotropy

There is, as almost in all other monogenic disorders, a variability of the phenotype in ARID cases. This variability is clear in enzyme defects, as residual activity of the enzyme due to hypomorphic variants may lead to milder phenotypes such as in galactosemia and the methylmalonic aciduria, but also for other phenotypes such as POLG and its associated disorders [13]. Furthermore, ARID genes may be pleiotropic, e. g., compound heterozygous pathogenic variants in the WD40 domain of *AHI1*, the main gene for Joubert syndrome, have been reported to lead to an isolated retinitis pigmentosa [14]. More glaring examples have been presented by several recent large publications, for example, Hu and colleagues describe two homozygous variants in the genes *AK1* and *ALS2* that lead to ARID [8]. *AK1* and *ALS2* are linked to hemolytic anemia due to adenylate kinase deficiency (OMIM#612631) and to juvenile amyotrophic lateral sclerosis 2 or ascending infantile onset spastic paralysis (OMIM#205100 and #607225) respectively. It seems that the pleiotropy of ARID genes has been underestimated. A major reason for this may be the small number of cases per gene that prohibit a delineated clinical description. Also, current strategies to identify novel ARID genes are not well-prepared for pleiotropy; if a gene has been associated with a different disorder, the investigator often assumes that there is no correlation. This can be overcome by changing the evaluation strategies and by running large, genome-wide meta-analyses that ignore pre-knowledge of gene–phenotype correlations.

## Research on the genetics of ARID

The first systematic study on the identification of genes in ARID was published by Najmabadi et al. [12]. Since then, a large number of studies that present one gene and several systematic but smaller studies have been published. In 2017 and 2018, five groups from Berlin and Tehran, Erlangen, Nijmegen, Toronto, and Riyadh [3, 6, 8, 16, 17] published landscape studies including more than 100 families each. Obviously, this was a reflection of the understanding that keeping the results unpublished has added value neither for the group nor for science. All five studies had very similar results: a) clarification rates were between 25 and 40%; b) high numbers of candidate genes were identified; c) the identified genes overlapped only a little; d) the identified genes and encoded proteins have plenty of cellular functions and are not specifically related to neurological functions. The recent evaluation of the DDD data regarding ARID led to the identification of only three novel genes [7].

Continuing this line of publishing, the detailed data of families who are examined in the future are required and would lead to further identification of ARID genes that are still unknown. Although the single evaluation of candidate genes identified in single families, followed by identifying further similar genotype–phenotype correlations and by functional analyses, would probably lead to delineation of the genetics of ARID, meta-analyses are also needed. The superordinate evaluation of thousands of families allows deviating presentations and pleiotropy to be identified and would enable recommendation regarding diagnostic and therapeutic strategies.

## Diagnostic strategies and recommendations

In Leipzig, we have performed 596 large panel diagnostics (4813 genes; TruSightOne, Illumina) in individuals with ID and have clarified an average of 29% of the cases. Performing trio-exome analysis led to the diagnosis of a further 22% of the cases. This, and the fact that for ARID (in addition to ADID and XLID) there is no significant cluster of mutations in a specific gene or gene group means that regardless of the inheritance mode and, in most of the cases, regardless of the symptoms of the affected individual, exome sequencing is an efficient and convenient diagnostic method for patients with ID in general and for identifying ARID causes specifically. However, panel analysis as a first diagnostic step is still an option at many centers, especially because the quality of sequencing is better in comparison to exomes. Owing to the variability in phenotypes, because even expert-curated lists of ID genes differ between centers, and because there are continual and fast additions to the literature, such panels do not cover the full spectrum. To our knowledge, there are no studies that show that a gene panel outperforms the yield of exome sequencing. In the end, such diagnostic strategies are a decision based on several factors.

## Practical conclusion

- Autosomal recessive forms of intellectual disabilities (ARID) contribute to about 10% of cases in an outbred population.
- In consanguineous families, the risk for ARID is a magnitude higher and the total risk for ID is about 2–3 times higher than for children of parents who are not related.
- Of presumably 2500–3000 ARID genes, less than 700 confirmed genes and less than 400 candidate genes have been identified.
- Of the undiagnosed causes of ID, 15–20% are suspected to be ARID.
- There are no particularly prevalent ARID genes or etiologies.
- Recently, a growing number of ARID genes have been reported to be pleiotropic. This and the variability in the phenotypic spectrum would enforce changes in diagnostic and research strategies.
- Diagnosing ID in general and ARID specifically is highly efficient via exome analysis. For ARID, there is rarely a justification for a targeted analysis.
